# Single midline incision approach for decompression of greater, lesser and third occipital nerves in migraine surgery

**DOI:** 10.1186/s12893-022-01675-z

**Published:** 2022-06-17

**Authors:** Danielle R. Olla, Kortni M. Kemper, Amanda L. Brown, Brian A. Mailey

**Affiliations:** 1 Southern Illinois University School of Medicine, Springfield, IL USA; 2 Institute for Plastic and Reconstructive Surgery, Department of Surgery, Southern Illinois University, 747 N. Rutledge St, Springfield, IL 62702 USA

**Keywords:** Migraine surgery, Occipital migraines, Greater occipital nerve, Lesser occipital nerve, Third occipital nerve, Single incision, Vertical incision

## Abstract

**Background:**

The traditional approach for occipital migraine surgery encompasses three separate surgical incisions in the posterior neck to decompress the greater occipital nerves (GON), lesser occipital nerves (LON), and third occipital nerves (TON). Other incisions have been investigated, including singular transverse incisions. We sought to evaluate a single, vertical midline incision approach for decompression of all six occipital nerves.

**Methods:**

Using 10 cadaveric hemi-sides (5 fresh cadaver head and necks). Anatomic landmarks and the location of the bilateral GON, LON, and TON were marked according to previous anatomic studies. A single, midline 9-cm incision was made, and lateral skin flaps were raised to decompress or avulse all six nerves.

**Results:**

Through the midline incision, the GON and TON were identified at 3.5 and 6.2 cm, respectively, inferior to a line bisecting the external auditory canal (EAC) and 1.5 cm lateral to the midline. The LON was identified as 6-cm inferior and 6.5-cm medial to a line bisecting the EAC in the plane just above the investing layer of the deep cervical fascia until the posterior border of the sternocleidomastoid was encountered. The LON had the greatest amount of variation but was identified lateral to the posterior border of the SCM.

**Conclusions:**

A single midline incision approach allows for successful identification and decompression of all six occipital nerves in migraine surgery.

## Background

Migraine headaches ranked as the third most prevalent disorder in the world in 2010 and the third-highest cause of disability worldwide in both males and females under the age of 50 in 2015 [1]. The pain of occipital migraines is located in the upper neck and posterior scalp region. These migraines may be associated with stress, posterior muscle tightness from exercise or whiplash trauma, and trigger point tenderness [2, 3]. Advancement in understanding the underlying pathophysiology has led to promising treatment modalities, including botulinum toxin A (BTX-A) injections and migraine surgery [4–8]. Screening the patient for surgery includes identifying their migraine trigger points, which can be assessed using pain sketches [9]. 

Traditional release of the nerves involved in occipital migraines requires three vertical incisions in the posterior neck. An approach with a transverse singular incision has been described, both with and without fat flaps, to insulate the fragile occipital nerves [10, 11]; however, our experience with this incision has limited ability to access superiorly enough to relieve entanglement of the GON and occipital artery. We investigated a singular midline, vertical incision to increase superior access to potential trigger points, while maintaining a single cosmetic incision.

Many anatomic studies have identified the compression points associated with occipital migraines, including the multiple points of compression of the greater occipital nerves (GON), the various zones of compression for the lesser occipital nerves (LON), and third occipital nerves (TON) (Table 1) [12–17]. Thorough understanding of the nerve locations and emergence points from under muscle and fascia is vital for successful treatment with BTX-A injections or surgical treatment (Table 2). In a systematic review, migraine headache surgery reported an average success rate of 90% with elimination of 50% or greater with an improvement of migraine headaches [18]. Sixty-two percent of patients with occipital migraine headaches reported total relief of migraine symptoms, and all patients had some element of improvement in migraine headaches after the open release of GON [5, 19].

**Table 1:** Compression points of occipital migraine headaches

GON	LON	TON
Musculofascial tissue surrounding obliquus capitus inferior muscle	Zone 1: Emergence from SCM	Point of exit from semispinalis muscle (multiple branches may be involved)
Epimysium underlying the semispinalis or the muscle itself	Zone 2: Posterior border of SCM	
Exit point from semispinalis muscle	Zone 3: Nuchal line crossing point (multiple branches may be involved)	
Insertion into the nuchal line		
Occipital artery		

*GON* greater occipital nerve, *LON* lesser occipital nerve, *TON* third occipital nerve, *SCM* sternocleidomastoid

**Table 2:** Location of previously defined sites of occipital nerves

	Distance from midline	Distance from line between auditory canals
Greater occipital nerve	1.5-cm	3.0-cm
Lesser occipital nerve	6.5-cm	5.3-cm
Third occipital nerve	1.3-cm	6.2-cm

Previously, occipital migraine surgery has been described with a 4.0-cm to 4.5-cm midline incision to address the GON and TON. Then, two separate incisions are placed laterally near the sternocleidomastoid (SCM) to address the LON. This leaves the patient with three incisions, increasing the risk of pain, wound breakdown, scarring, neuroma formation, and unsatisfactory aesthetic appearance of the posterior scalp and neck. This study describes an approach to occipital migraine surgery with a single, vertical, midline incision to safely decompress the GON, TON, and the more laterally located LON.

## Methods

**Fig. 1:**
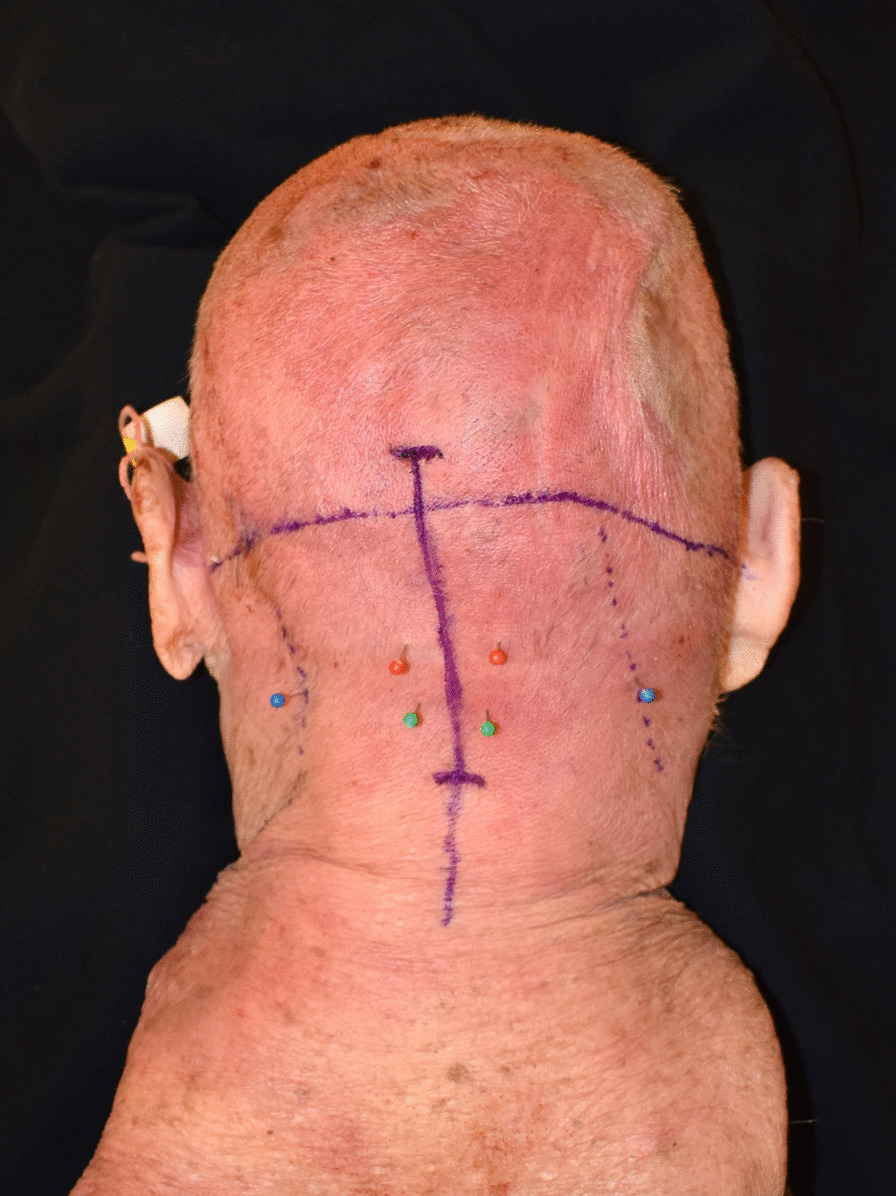
Marked 8-cm midline incision in the caudal occipital region down into the superior neck. This extended incision was used to demonstrate the landmarks in this cadaveric specimen. A more limited 5-cm incision is typically sufficient to access all of the nerves in vivo. Marked anatomic location of GON 1.5-cm from the midline and 3.5-cm from the EAC (red), LON 6.5-cm from midline and 6-cm from the EAC (blue), TON 1.3-cm from midline and 6.2-cm from the EAC (green)

Ten cadaveric hemi-sides were provided by Southern Illinois University. The cadaveric head was placed in the prone position. The posterior border of the SCM was marked bilaterally. A line was then drawn in the horizontal plane at the level of the external auditory canal (EAC) followed by a line vertically down midline from level of occipital protuberance to the base of the neck. The GONs were marked 1.5-cm from the midline and 3.5-cm from the EAC. The LONs were marked 6.5-cm from midline and 6-cm from the EAC. The TONs were marked 1.3-cm from midline and 6.2-cm from the EAC [12]. We then designed a 9-cm long midline incision in the caudal occipital region down into the superior neck (Fig. 1). An extended incision was used to demonstrate the landmarks in this cadaveric specimen; however, a more limited 5-cm incision is typically sufficient to access all of the nerves in vivo.

The incision was made through the skin and subcutaneous tissue down to the midline raphe. Large subcutaneous skin flaps were raised laterally, just above the investing layer of the deep cervical fascia. The dissection was continued with spreading technique to identify the LON along the posterior border of the SCM in the subcutaneous plane. Once the LON was identified, it was followed superiorly to confirm its identity and avulsed, or sites of compression were released until the nerve entered the subcutaneous tissue.

**Fig. 2:**
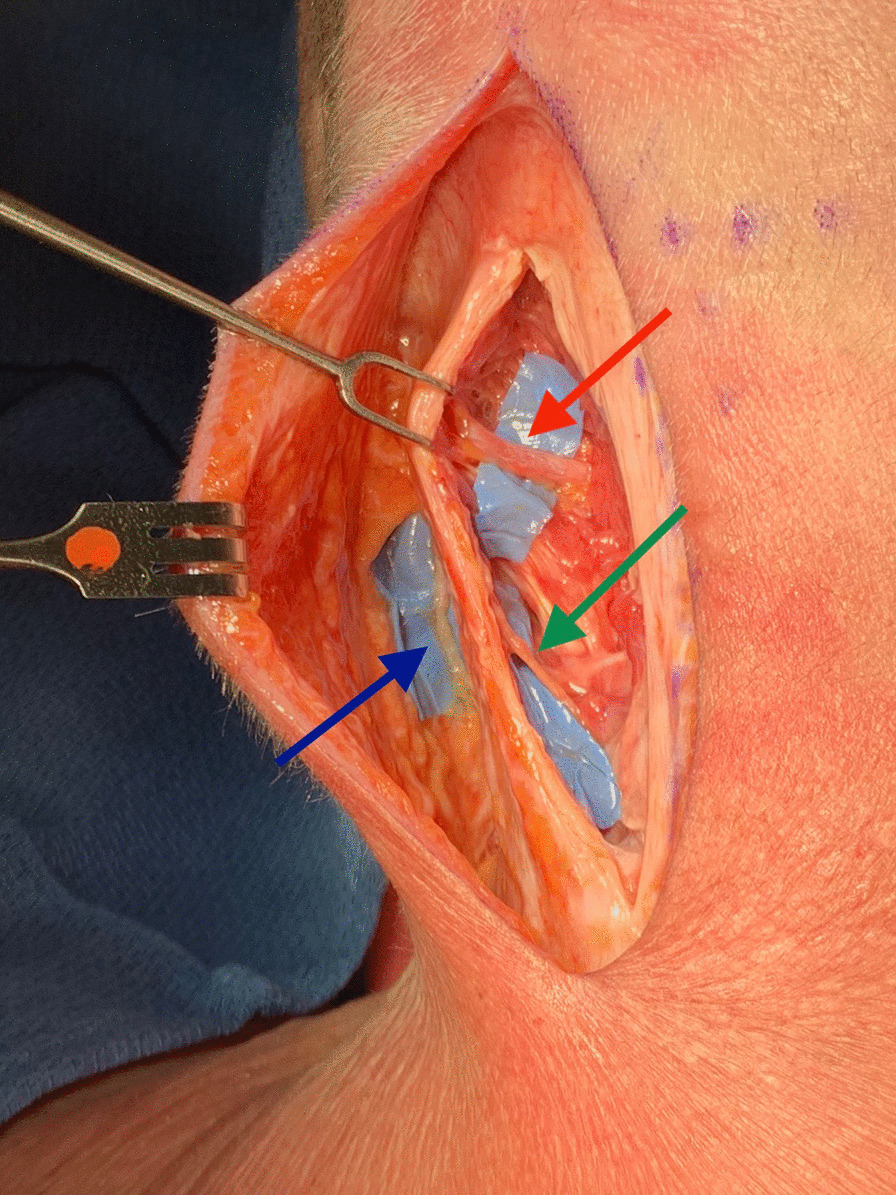
Lateral skin flap raised just above the investing layer of the deep cervical fascia with exposed GON (red arrow), LON (blue arrow), TON (green arrow)

**Fig. 3:**
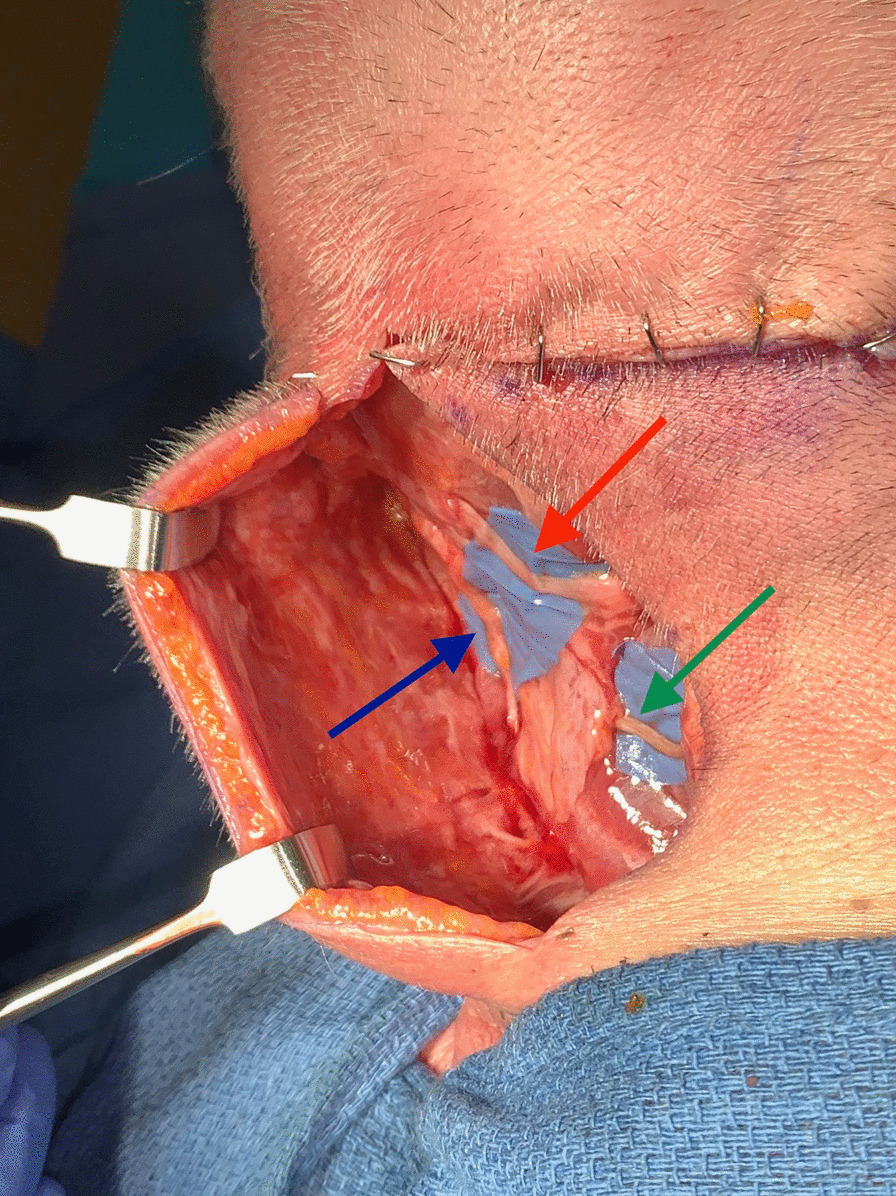
Exposed GON (red arrow), LON (blue arrow), TON (green arrow) through single midline incision. The transverse incision was created to aid in the dissection and to demonstrate the anatomy

After addressing the LON through a more superficial plane, the deeper dissection for the GON began. The trapezius fascia was incised 0.5-cm lateral to midline leaving the midline raphe intact. When present, the oblique trapezius muscle was retracted laterally. The semispinalis capitus was found just below the fascia running in the vertical direction and dissection was carried subfascially until the trunk of GON was identified. Then, 2.5-cm of the semispinalis muscles medial to nerve was excised. Each compression point was released as the nerve was followed distally to its entrance to the subcutaneous tissue. During the release of the GON, the TON was encountered a similar distance from midline but inferior to the GON. It was avulsed as its sensation contributions are small (Figs. 2 and 3).

## Results

Ten cadaveric hemi-sides were dissected, and each nerve was sequentially identified. The GON and TON were consistently located at their anatomic landmarks. The LON had more location variation and could be challenging to identify in some instances. The most common dissecting error encountered was being in a deeper plane than the SCM. The posterior border of the SCM lies deep to the subcutaneous tissues of the neck. The LON is easiest to identify emerging posterior to the SCM muscle in the subcutaneous plane and coursing upwards towards the occiput. The great auricular nerve (GAN) and spinal accessory nerve (SAN) can be encountered in this area, but course differently than the LON. The GAN and SAN also emerge posterior to the SCM, but the GAN courses more anterior than the LON, while the SAN runs inferiorly and obliquely to the LON and varies in caliber from 1 to 4 mm in size [20–22]. Following the LON to the superior posterior occiput can help confirm its identity. A nerve stimulator can also help confirm if motor fibers are present.

After each nerve was dissected and photographs were taken, the skin flaps were retracted to follow the nerves' course to confirm their identities further. The LON was challenging to locate in two (4 hemi-sides) of the cadaver heads requiring more extensive and prolonged dissection. Keeping the dissection plane superficial to the SCM helped maintain landmarks and ease the identification of the nerves. The dissection plane created from the midline only requires skin flap elevation of 3–4 additional centimeters on each side and can provide a broader perspective on the location of each nerve.

## Discussion

Traditionally, occipital migraine surgery is performed with three separate incisions to decompress the GON, TON, and LON. Approaches with a transverse, singular incision have been described [10, 11]; however, this incision may have limited superior access intraoperatively, leading to the incomplete release of the GON from the occipital artery. We investigated a singular midline, vertical incision to increase superior access to potential trigger points of occipital migraine headaches. The location of occipital artery involvement around the GON can be identified preoperatively via doppler ultrasound [23]. This method allows for quick evaluation and assistance to the surgeon in deciding which incision is most indicated.

More incisions can increase pain, neuroma formation, and risk of wound breakdown. Scars on the back tend to widen, creating concern for the aesthetic appearance of the scars. We have successfully performed a vertical, single incision release of all six nerves in six hemi-necks. The well-defined anatomic locations of the GON, LON, and TON make this single vertical incision approach a safe and feasible option. We used up to a 9-cm incision for the cadaver dissection; however, a shorter incision may be made in vivo if the patient's trigger points can be accessed. We find this approach to be ideal for smaller necks but will use longer incisions or the more traditional 3-incision approach in larger necks.

Creating large skin flaps is essential to achieving adequate exposure. The skin flaps should be elevated just above the fascia to preserve the musculature and fascial planes. The fascia should be entered just at the posterior border of the SCM to locate the LON. It is also important to note the course of the LON as it emerges from the posterior aspect of the SCM and then travels on the anterior surface of the SCM. The GAN emerges just inferior and follows a similar course [24]. The TON is close to the GON, located just inferior as it pierces the semispinalis muscle, but is smaller in caliber and can be easily missed.

Seroma can develop with a larger dissection plane. Due to the dead space, use of a drain may help reduce seroma formation. We occasionally also utilize a drain in the traditional 3-incision approach.

## Conclusions

Occipital migraine surgery has been established as a beneficial treatment option but traditionally requires three separate incisions to release the paired GON, LON, and TON. These six nerves can all be accessed and released through a single midline incision. The course of the LON has the most variation and is the most challenging to locate; however, the wider midline approach can provide an anatomic perspective for successful identification of each nerve while preserving the planes and musculature in the posterior neck. The vertical incision accommodates reaching the superior entanglement points of the occipital artery around the GON. Overall, we have found this vertical central incision allows access to the occipital nerves, especially in smaller necks. A single midline incision is well concealed and is located mostly in the hairline. Traditional incisions are more visible and may not always be necessary.

## Data Availability

Not applicable.

## Abbreviations

GON: Greater occipital nerve
LON: Lesser occipital nerve
TON: Third occipital nerve
EAC: External auditory canal
SAN: Spinal accessory nerve
GAN: Great auricular nerve
SCM: Sternoscleidomastoid muscle
BTX-A: Botulinum toxin A
